# Complete genome sequencing and analysis of endophytic *Sphingomonas* sp. LK11 and its potential in plant growth

**DOI:** 10.1007/s13205-018-1403-z

**Published:** 2018-08-28

**Authors:** Sajjad Asaf, Abdul Latif Khan, Muhammad Aaqil Khan, Ahmed Al-Harrasi, In-Jung Lee

**Affiliations:** 1 Natural and Medical Sciences Research Center, University of Nizwa, 616 Nizwa, Oman; 2 School of Applied Biosciences, Kyungpook National University, Daegu, 41566 Republic of Korea

**Keywords:** *Sphingomonas* sp. LK11, Endophyte, Plant growth promotion, Genome, SMRT sequencing

## Abstract

Our study aimed to elucidate the plant growth-promoting characteristics and the structure and composition of *Sphingomonas* sp. LK11 genome using the single molecule real-time (SMRT) sequencing technology of Pacific Biosciences. The results revealed that LK11 produces different types of gibberellins (GAs) in pure culture and significantly improves soybean plant growth by influencing endogenous GAs compared with non-inoculated control plants. Detailed genomic analyses revealed that the *Sphingomonas* sp. LK11 genome consists of a circular chromosome (3.78 Mbp; 66.2% G+C content) and two circular plasmids (122,975 bps and 34,160 bps; 63 and 65% G+C content, respectively). Annotation showed that the LK11 genome consists of 3656 protein-coding genes, 59 tRNAs, and 4 complete rRNA operons. Functional analyses predicted that LK11 encodes genes for phosphate solubilization and nitrate/nitrite ammonification, which are beneficial for promoting plant growth. Genes for production of catalases, superoxide dismutase, and peroxidases that confer resistance to oxidative stress in plants were also identified in LK11. Moreover, genes for trehalose and glycine betaine biosynthesis were also found in LK11 genome. Similarly, *Sphingomonas* spp. analysis revealed an open pan-genome and a total of 8507 genes were identified in the *Sphingomonas* spp. pan-genome and about 1356 orthologous genes were found to comprise the core genome. However, the number of genomes analyzed was not enough to describe complete gene sets. Our findings indicated that the genetic makeup of *Sphingomonas* sp. LK11 can be utilized as an eco-friendly bioresource for cleaning contaminated sites and promoting growth of plants confronted with environmental perturbations.

**Electronic supplementary material:**

The online version of this article (10.1007/s13205-018-1403-z) contains supplementary material, which is available to authorized users.

## Introduction

Endophytic microorganisms, specifically bacteria or fungi, are known to inhabit plant tissues without causing disease symptoms in the host plant (Hallmann et al. 1997; Reissinger et al. 2001; Wilson 1995). Endophytic microbial communities have vital roles in the development and growth of various host plants under favorable and various stress conditions, such as heat, salinity, heavy metal contamination, and drought (Yaish et al. 2015). Among endophytes, bacteria have a knack for inhabiting internal plant tissues and imparting beneficial effects for host growth. Such traits have been shown to improve growth and developmental processes (Glick 1995; Ryan et al. 2008) of the host through the ability of endophytes to perform a range of functions, including assisting both primary and secondary nutrient uptake via atmospheric nitrogen fixation (Gothwal et al. 2008), synthesizing iron siderophores (Wang et al. 1993), and solubilizing minerals such as phosphate, potassium, and zinc (Basak and Biswas 2009; Iqbal et al. 2010; Kang et al. 2009). Facilitation of plant growth promotion by endophytic bacteria occurs through several mechanisms; these include mineralization of inorganic substances from the soil into host roots and production of enzymes, phytohormones, and defense-related constituents within the host environment (Khan et al. 2016a; Santoyo et al. 2016). In addition, these endophytic microbes can support the plant by providing nitrogen sources (by fixing atmospheric nitrogen into ammonia) and other nutrients, such as sulfur, iron, and phosphate. Furthermore, these microbes can protect their host plants from pathogenic attacks by regulating host plant physiology and phytohormones (Bach et al. 2016).

The endophytic bacterium *Sphingomonas* sp. LK11 was first isolated from the leaves of the arid medicinal plant *Tephrosia apollinea* and was subsequently found to actively increase growth and stress tolerance in tomato plants during salinity and cadmium stress (Halo et al. 2015; Khan et al. 2014). It has also been suggested that LK11 can produce phytohormones such as gibberellins (GAs) and auxins (Khan et al. 2014). Members of the genus *Sphingomonas* are yellow-pigmented, rod-shaped, nonsporulating, Gram-negative, chemoheterotrophic, and aerobic bacteria that belong to class Alphaproteobacteria within the phylum Proteobacteria (Busse et al. 2003). *Sphingomonas* species have been isolated from several different environments; novel strains have recently been isolated from abandoned heavy metal sites (Feng et al. 2014), forest soil (Kim et al. 2014), indoor air of pharmaceutical environments (Park et al. 2015), purplish paddy soil (Huang et al. 2014), glaciers (Miteva et al. 2004), volcano-associated lakes (Farias et al. 2011), space shuttles (Pan et al. 2016b), permafrost (Piao et al. 2016), and the sediment of a eutrophic reservoir (Huy et al. 2014). However, there are few reports describing *Sphingomonas* species as endophytes.

*Sphingomonas* species have been mostly described regarding their roles in remediating or degrading various kinds of organic and inorganic pollutants from different contamination sources. Similarly, the LK11 strain can reduce Cd^2+^ uptake, accumulate intracellular Zn^2+^, and increase metallothionein expression (which excludes heavy metals and prevents their binding by related proteins) in their host plants (Khan et al. 2014). This endophyte has the potential to thrive in high salinity (contaminated with sodium chloride) without utilizing its cellular mechanisms for producing antioxidants and related enzymes, such as peroxidases (PODs), polyphenol oxidases (PPOs), and catalases (CATs) (Halo et al. 2015). Furthermore, LK11 was recently reported to improve plant growth in both wild type and Got-3 mutant tomato plants when exogenously introduced to the plants via jasmonic acid (JA) treatment (Khan et al. 2017). The combined effects of LK11 and JA treatment caused plants to respond positively to salinity stressors by dramatically regulating glutathione content in Got-3 mutant and wild type tomato plants (Khan et al. 2017). Recent studies have also demonstrated the role of *Sphingomonas* spp. in the degradation of organic chemical compounds, such as bisphenol (Fujiwara et al. 2016), phenol (Gong et al. 2016), triclocarban (Mulla et al. 2016), phenanthrene (Liu et al. 2016), chlorogenic acid (Ma et al. 2016), nonylphenol polyethoxylates (Bai et al. 2016), astaxanthin (Ma et al. 2016), dioxin (Miller et al. 2010), γ-hexachlorocyclohexane (Tabata et al. 2013), nicotine (Zhu et al. 2016), plasticizers (Kera et al. 2016), and hexachlorocyclohexane isomers (Kumari et al. 2002) among others. In addition to these degradation abilities, the *Sphingomonas* genus can also produce bioactive metabolites, such as indole acetic acid, gibberellins, sphingan (Li et al. 2016), and gellan gum (Gai et al. 2011b).

Previous studies have suggested the potential of LK11 as a plant growth-promoting bacterium; however, this strain has not been fully investigated for these characteristics. Therefore, the current study aimed to elucidate the whole LK11 genome and its plant growth-promoting activity. Sequencing the complete genome of LK11 will aid in resolving the complex biological mechanisms of this microorganism that promote plant growth and induce hardiness against salinity and heavy metal stress. These genomic analyses will provide a foundation towards fully understanding the characteristics of this microorganism and its potential for broader application against environmental stressors. Furthermore, comparisons with other completely sequenced *Sphingomonas* genomes will help delineating the unique and shared traits among different *Sphingomonas* species, offering insights into the evolutionary changes that have occurred within this genus.

## Materials and methods

### Detection of gibberellins (GAs) in cell-free cultures

*Sphingomonas* sp. LK11 was cultured in NB media and incubated for 7 days at 30 °C and 200 rpm. Quantification of GA in bacterial cultures was carried out according to the protocol described by Kang et al. (2016) and Waqas et al. (2012). Bacterial culture filtrates supplemented with [^2^H_2_] GA standards were processed for detection, identification, and quantification of GA using gas chromatography and mass spectroscopy.

### *Sphingomonas* sp. LK11-plant interaction

Healthy soybean seeds were obtained from the Soybean Genetic Resource Center (Kyungpook National University, Daegu, South Korea) with a 95% germination rate. Surface sterilization and germination experiments were carried out according to Asaf et al. (2017b). Sterilized germination trays and pots were filled with horticulture soil that had been autoclaved (121 °C and 15 psi for 15 min) three times and had the nutrient composition of peat moss (Asaf et al. 2016). After germination, randomly selected uniform plant seedlings were planted in one round plastic pot (10 × 9 cm) and grown for 20 days using one of two treatments, (1) control plants without LK11 or (2) plants inoculated with LK11. Distilled water was applied to plants as needed and with care to prevent leaching. LK11 cells dissolved in 35-mL sterilized double-distilled water were applied three times to treatment (1) plants to ensure efficient transformation and then twice consecutively at 1-week intervals. Endophyte cells were collected as described above. The harvested cells were then washed with 0.8% NaCl solution and dissolved in autoclaved double-distilled water adjusted to an optical density (OD) of 0.5. Different plant physiological parameters like shoot length, root length, and fresh and dry weight were analyzed. Furthermore, plants were transferred to liquid nitrogen and freeze-dried for 1 week using a freeze dryer (VirTis, Gardiner, NY, USA) for GA analysis.

### Quantification of endogenous GAs in soybeans treated with LK11

Quantification of GAs in the freeze-dried samples of soybean plants was carried out according to the protocol established by Lee et al. (1998) using gas chromatography with a mass spectrometer (6890N Network GC system and 5973 Network Mass Selective Detector; Agilent Technologies). The results were calculated in ng/gof freeze-dried weight of plant samples.

### DNA extraction, genome sequencing, and genome assembly

*Sphingomonas* sp. LK11 was previously isolated and identified by Khan et al. (2014). For complete genome sequencing, genomic DNA of LK11 was extracted from an overnight cell suspension culture using the Qiagen™ QIAamp DNA Mini Kit (Qiagen, Hilden, Germany). Complete genome sequencing was performed using the Single Molecule Real Time (SMRT) sequencing technology of Pacific Biosciences (PacBio, Menlo Park, CA, USA) as described previously (Chan et al. 2014). Briefly, a PacBio large insert library (15–20 kb) was constructed from high molecular DNA (120.0 ng/µL) and sequenced on four V2 SMRT cells using P4-C2 chemistry with a running movie for 4 h at the Duke Center for Genome and Computational Biology, Duke University (Durham, NC, USA). PacBio produces data in HDF5 format (*.h5) and the corresponding input file of SMRT Analysis software is a bas.h5 file or an associated bax.h5 file. Assemblies were evaluated to ensure data quality using QUAST 2.3 (Gurevich et al. 2013). A total of 84,384 reads, with a mean read length of 11,888 bp, was generated. The reads were de novo assembled into a circular chromosome and two circular plasmids, with an average genomic coverage of 150.26 reads (Table S1), using the Hierarchical Genome Assembly Process (HGAP) workflow in SMRT Portal (version 2.1.1).

### Genome annotation

Complete genome annotation was performed using the NCBI Prokaryotic Genome Annotation Pipeline (Angiuoli et al. 2008). This annotation was used to predict coding genes through an *ab initio* gene prediction algorithm with homology-based methods. The annotation process helped elucidate functional genomic units, such as structural RNAs (5S, 16S, and 23S), tRNAs, and small noncoding RNAs. Additional gene prediction analysis and functional annotation were performed by Rapid Annotation using Subsystem Technology (RAST) version 3.0 (Aziz et al. 2008a, b; Brettin et al. 2015; Overbeek et al. 2014) and the Integrated Microbial Genomes platform (IMG) (Markowitz et al. 2012). The assembled and annotated sequences of LK11 (one chromosome and two plasmids) were deposited in GenBank with accession numbers CP013916–CP013918. This information was submitted to the Genomes Online Database (Gs0118031) (Reddy et al. 2015).

### Comparative genome analysis

To understand the genomic features of *Sphingomonas* sp. LK11 (CP013916), comparative assessments were made with the recently reported genome sequences of *Sphingomonas* sp. MM1 [CP004036; (Tabata et al. 2013)], *Sphingomonas* sp. NIC1 [CP015521; (Zhu et al. 2016)], *Sphingomonas taxi* [CP009571; (Eevers et al. 2015)], and *Sphingomonas hengshuiensis* [CP010836; (Wei et al. 2015)]—all of which were obtained from NCBI. Gene prediction and functional annotation of these *Sphingomonas* spp. were performed using the RAST subsystem (Aziz et al. 2008b; Brettin et al. 2015; Overbeek et al. 2014). For comparison purposes, we created a circular genomic map of each genome using Interactive Microbial Genome Visualization with GView (Petkau et al. 2010) and Ring Image Generator (BRIG, version 0.95) (Alikhan et al. 2011). Each circular genomic map was generated with BLAST+, with standard parameters (70% lower and 90% upper cutoff for identity and *E* value of 10), using the LK11 genome as the “alignment reference genome.” Pan-genome and core genome analyses of LK11 against related species were carried out using EDGAR version 2.0 (Blom et al. 2009) and PGAP version 1.12 (Zhao et al. 2012).

## Results and discussion

### Plant growth-promoting traits of *Sphingomonas* sp. LK11

**Fig. 1:**
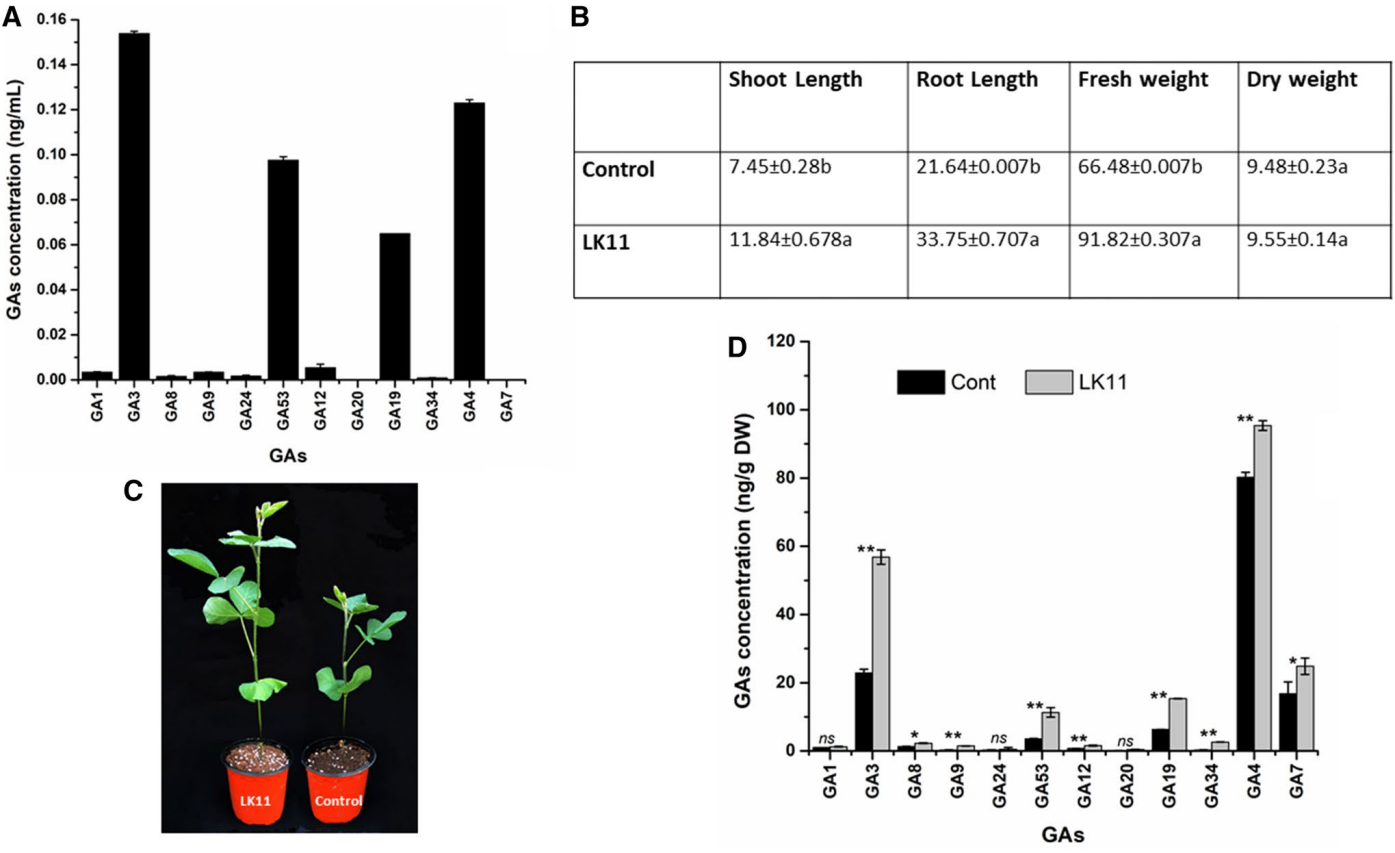
Gibberellin (GA) production by *Sphingomonas* sp. LK11 (a). Bacterial culture was centrifuged and 100 mL of the culture filtrate was analyzed for the presence of GAs using a GA extraction protocol. The bar indicates standard deviation between replicates. Effect of *Sphingomonas* sp. LK11 culture on (**b, c**) different growth attributes and (**d**) endogenous GA of soybean plants. Same letters indicate non-significant difference within treatment, while (*) and (**) indicate significant and very significant differences, respectively. (ns) represents non-significant difference among different types of GAs

The results showed that LK11 produces different quantities of GAs in its pure culture; these included GA_1_, GA_3_, GA_8_, GA_9_, GA_24_, GA_53_, GA_12_, GA_20_, GA_19_, GA_34_, GA_4_, and GA_7_ (Fig. 1a). Among these, physiologically active GA3 and GA4 were produced in significantly high quantities while inactive GA_53_ and GA_19_ were abundant in the pure culture of *Sphingomonas* sp. LK11. Other GAs were present in very small quantities (Fig. 1a). This is in agreement with a previous report by Khan et al. (2014) on the production of GA in pure culture; however, we found increased abundance of other GAs, such as GA_1_, GA_3_, GA_8_, GA_24_, GA_53_, GA_12_, GA_20_, GA_19_, and GA_34_, which is reported for the first time in the LK11 strain. Previous studies have shown that some bacterial strains also produce _GA_s, e.g., *Rhizobium phaseoli* (Atzorn et al. 1988), *Acetobacter diazotrophicus* (Bastián et al. 1998), *B. licheniformis* (Gutiérrez-Mañero et al. 2001), *B. cepacia* SE4 (Kang et al. 2014), *Leifsonia xyli* SE134 (Kang et al. 2017), and *Bacillus amyloliquefaciens* RWL-1 (Shahzad et al. 2017).

Since *Sphingomonas* sp. LK11 produces GAs, we examined the plant growth-promoting potential by inoculating soybean plants with pure LK11 culture. The results showed that *Sphingomonas* sp. LK11 significantly increased shoot, root, and plant biomass compared with control plants (Fig. 1b). This was further validated by changes in endogenous GA content of soybean plants. GA_3_ (88.2%), GA_7_ (8.2%), and GA_4_ (23.8%) were significantly higher in LK11-inoculated soybean plants than in control plants (Fig. 1d). Nagel and Peters (2017) suggested that bacterial strains possess active GA biosynthesis pathways as well as GA_4_ and GA_9_. Furthermore, such plant growth-promoting effects have been previously suggested due to the potential of microbes in producing phytohormone-like compounds (Khan et al. 2015). It has been reported that *Sphingomonas* sp. LK11 improves tomato plant growth (Khan et al. 2014), which is consistent with studies by Xu et al. (1998), Cerny-Koenig et al. (2005), Kang et al. (2014), and Shahzad et al. (2017), which showed that GA-producing bacteria are beneficial for improving crop growth. In addition, utilization of bacteria isolated from arid land ecosystems is more compatible with improving plant growth during harsh environmental conditions (Asaf et al. 2017b). Due to the ecological importance of such strains, we performed whole-genome sequencing of *Sphingomonas* sp. LK11.

### *Sphingomonas* sp. LK11 genome in comparison with related species

**Fig. 2:**
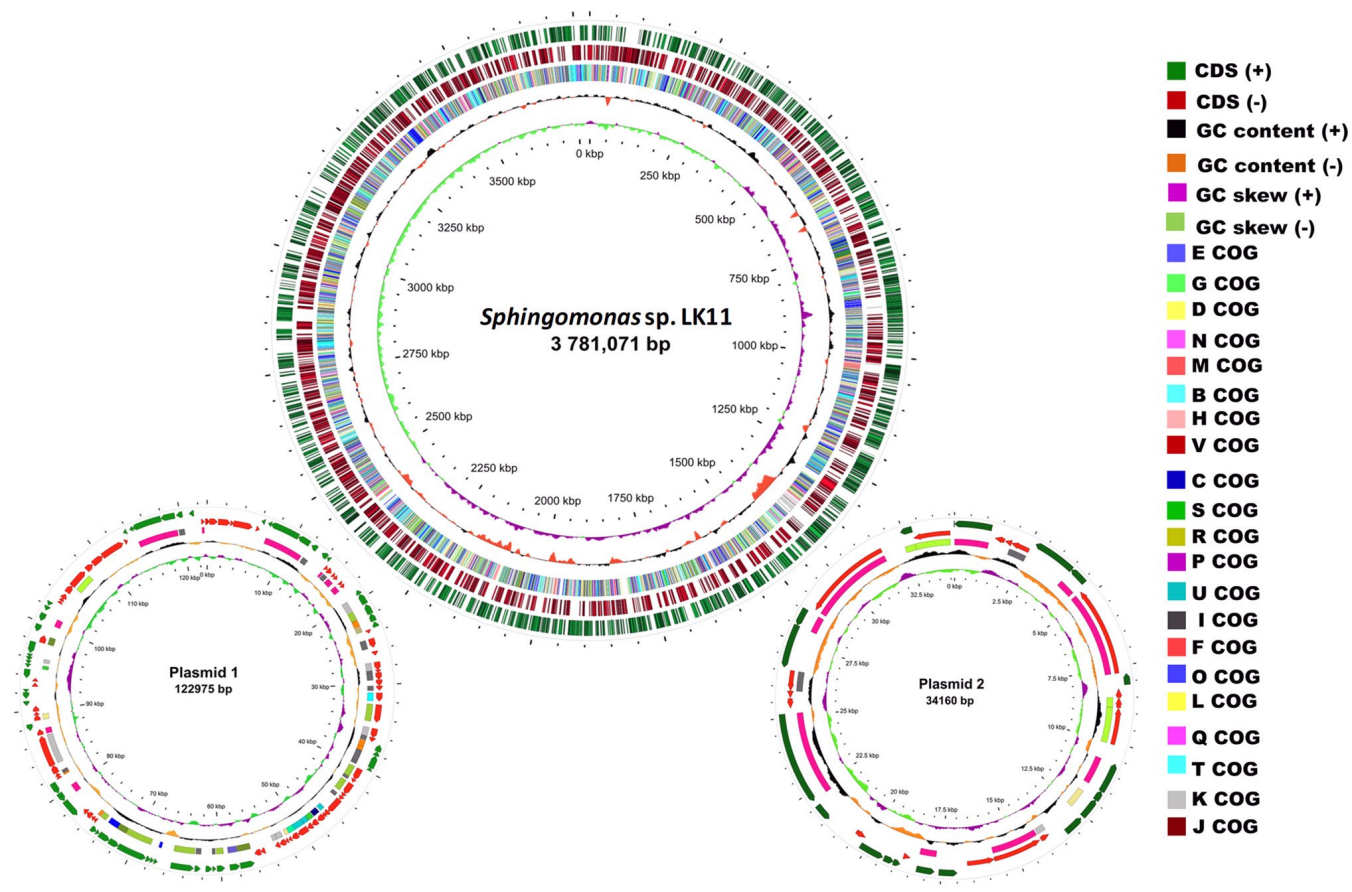
Circular representation of the *Sphingomonas* sp. LK11 genome. From outer to inner circles, the two outer circles show the predicted protein-coding sequences on the plus (green) and minus (red) strand. The third circle shows the distribution of genes related to Clusters of Orthologous Groups (COGs) categories, while the fourth and fifth circles show G+C content and G+C skew, respectively

The complete genome of *Sphingomonas* sp. LK11 was found to consist of a 3781,071 bp circular chromosome with a G+C content of 66.2% and two circular plasmids of 122,975 bp and 34,160 bp with G+C contents of 63 and 65%, respectively (Fig. 2; Table 1). When combined, the chromosome and plasmids contained 3739 annotated genes, including 59 tRNAs, 4 complete rRNA, and 3656 protein-coding sequences (CDSs; Table 1). Among these CDSs, 2388 (63.87%) genes were classified into clusters of orthologous group (COG) families comprised of 23 categories (Table S2). The genome size of LK11 falls within the expected range (based on other genomic studies) and a varying number of plasmid has been observed in other strains (Gai et al. 2011a; Kera et al. 2016; Li et al. 2016; Miller et al. 2010; Pan et al. 2016a).

**Table 1:** Gene prediction and annotation summary

Annotation statistics	
Genome size (bp)	3,938,206
GC	66.05
Total number of genes	3739
Number of CDSs	3656
Pseudogenes	114
rRNA genes	12
tRNA genes	59
Protein-coding genes with function prediction	2785
Protein-coding genes without function prediction	871
Protein-coding genes encoding enzymes	1001
Protein coding genes connected to KEGG pathways	1076
Protein coding genes connected to KEGG Orthology (KO)	1852
Protein coding genes connected to MetaCyc pathways	876
Protein coding genes with COGs	2388

Based on the diverse functional roles of species belonging to genus *Sphingomonas*, 2785 (74.49%) LK11 genes were assigned specific biological roles; this was also based on results from BLASTn homology searches. The remaining CDSs were categorized as proteins with unknown functions. Proteins, rRNAs, and tRNAs are encoded by 88.59%, 0.52%, and 0.121% of the complete genome, respectively, while the remaining 10.76% of the genome is made up of noncoding regions.

### Plant growth-promoting potential of *Sphingomonas* sp. LK11

From the genomic sequence of *Sphingomonas* sp. LK11, we analyzed genes that are categorized by their ability to enhance nutrient availability, catabolize aromatic compounds, and resist oxidative and other forms of abiotic stress (Table 2). Very few *Sphingomonas* species are reported to stimulate plant growth through the production of phytohormones or enzymes (Dodd et al. 2010). On the other hand, LK11 was shown to enhance plant growth through the production of GAs (Fig. 1b, c) and IAA (Khan et al. 2014). However, a complete IAA biosynthetic pathway was not found in LK11 during genome analysis, although some genes responsible for IAA production, such as the tryptophan biosynthesis gene cluster (*trpA, trpB*, and *trpD*) and indole pyruvate ferredoxin oxidoreductase (IOR; locus AV944_07715 and locus AV944_07710, respectively) were present. It has been well-established that the presence of tryptophan-related genes in bacterial genomes is linked to IAA biosynthesis and related biological functions (Gupta et al. 2014; Tadra-Sfeir et al. 2011).

**Table 2:** Genes attributed to plant growth promoting traits in the LK11 genome

Plant growth promotion traits	Genes with potential for PGP traits
Phosphate metabolism	*pstC, pstA, phoU, phoQ, nad(P), phoR* (*sphS*), *phoB, pstB, oprO, pstS*
IAA production	Tryptophan synthase α chain (*trpA*), Anthranilate phosphoribosyltransferase (*trpD*), Tryptophan synthase β chain (*trpB*), Phosphoribosylanthranilate isomerase (*PAI*)
Trehalose metabolism	trehalose synthase gene homolog
Chitinase	Chitinase gene homolog
H2S Production	*cysP, cysW, cysT, cysA*
Heat shock proteins	*dnaK, hrcA, dnaJ, rpoH, lepA, rdqB, smpB, grpE*
Cold shock proteins	*cspA, cspB*
Superoxide dismutase	Superoxide dismutase gene homologs
Sulfur assimilation	*cysT, cysW, cysP, cysA, cysQ, cysX, sat1, sat 2*

The LK11 genome also encodes cystathionine γ-lyase (CSE; locus AV944_16960), 3-mercaptopyruvate sulfurtransferase (3MST; locus AV944_12370), cystathionine β-synthase (CBS), and cysteine aminotransferase (CAT; locus AV944_01390), which are known for hydrogen sulfide (H_2_S) production. H_2_S production by plant growth-promoting rhizobacteria (PGPR) has been reported to enhance plant growth, seed germination, and root colonization (Dooley et al. 2013). The presence of an ATP-binding cassette (ABC) transporter that includes periplasmic binding proteins encoded by *cysP, cysT, cysW*, and *cysA* in the LK11 genome revealed that these genes may be involved in the transportation of thiosulfate or inorganic sulfate to cells as reported earlier in *Pseudomonas* sp. UW4 (Duan et al. 2013). The presence of these genes in bacterial strains has been linked to oxidation of sulfur and sulfur-conjugated metabolites (Kwak et al. 2014). Moreover, sulfur oxidation influences soil pH and sequentially improves solubility of micronutrients, such as N, P, K, Mg, and Zn (Vidyalakshmi et al. 2009). Therefore, the association of such endophytic microbes can provide improved mineral acquisition and allocation to the host plants.

We also identified glucose-1-dehydrogenase (*gcd*; locus AV944_13915) in the LK11 genome, suggesting that LK11 can solubilize inorganic mineral phosphates, making it a potential inoculant candidate for increasing phosphorous uptake in plants. Some bacteria were reported to solubilize insoluble mineral phosphates by producing organic acids (mainly gluconic acid) and acid phosphatases (Achal et al. 2007), where the production of gluconic acid is assisted by *gcd* (de Werra et al. 2009). Inorganic phosphates are important for plant growth and thus microbes can assist plants by mobilizing complex phosphates into more solubilized forms (Gupta et al. 2012). Several bacteria such as *Gluconobacter oxydans, Pseudomonas fluorescens, Azospirillum* spp., and *Mesorhizobium mediterraneum* have shown phosphate-solubilizing abilities (de Werra et al. 2009; Peix et al. 2001; Rodriguez et al. 2004).

In addition to *gcd*, the phosphate-specific transport (*pst*) system is used for free inorganic phosphate transport in *Bacillus subtilis* and *Escherichia coli*. The *pst* operon of *E. coli and B. subtilis* is composed of *pstS, pstC, pstA*, and *pstB* as well as a two-component signal transduction system consisting of *phoP*/*phoR* for phosphate uptake (Xie et al. 2016). In the present study, genomic analyses of LK11 revealed that it also carries the *pst* operon (*pstA, pstB, pstC*, and *pstS* genes; locus AV944_10605, locus AV944_10610, locus AV944_10600, and locus AV944_10615, respectively), as well as *phoB* (locus AV944_10590), *phoP* (locus AV944_05370), and *phoR* (locus AV944_10620) genes for phosphate transport.

### *Sphingomonas* sp. LK11 in osmotic stress

Plants are often exposed to abiotic stresses such as heat, drought, metal contamination, and high salinity. In such circumstances, inoculating plants with symbiotic, stress-regulating microbes can provide them with additional means of combating stress conditions (Khan et al. 2015; Yang et al. 2009). Abiotic stresses can create osmotic deficiencies in plant cells, while microorganisms in the phyllosphere can produce extracellular polysaccharides to protect not only themselves but their plant hosts from adverse effects (Beattie and Lindow 1999). Recently, *Sphingomonas* sp. LK11 was reported to significantly increase plant height, biomass, and glutathione, amino acid, and primary sugar levels compared with control under varying drought stresses (Asaf et al. 2017a). These findings were further validated by the presence of trehalose biosynthesis pathways (*otsA*/*otsB* and *treY*/*treZ*) in the genome of LK11. Trehalose can act as an osmoprotectant and the *otsA*/*otsB* pathway is considered the most widely occurring biochemical pathway in many organisms that are under environmental stressors, such as high salinity, drought, low temperature, and osmotic stress (Duan et al. 2013; Garg et al. 2002). Moreover, trehalose production protects microbes from oxidative stress, including exposure to hydrogen peroxide (Pilonieta et al. 2012). This is supported by a recent study where exogenous trehalose and *Sphingomonas* sp. LK11 inoculation of soybean plants significantly mitigated polyethylene glycol-induced drought stress through activating endogenous primary sugars (Asaf et al. 2017a). The presence of these trehalose pathways in the LK11 genome suggests that this strain can aromatic hydrocarbons. It has also been demonstrated that trehalose accumulation may act as a biosurfactant that enhances biodegradation of hexachlorocyclohexane, which was previously reported for *Sphingomonas* sp. NM05 (Garg et al. 2002; Manickam et al. 2012).

In addition, the LK11 genome was found to contain a number of salt tolerance genes that can synthesize the osmolyte glycine betaine from choline by encoding the *betT* choline transporter (Lamark et al. 1996), the *betA* choline dehydrogenase, and the *betB* betaine aldehyde dehydrogenase. The presence of these genes further validates our recent findings related to the role of LK11 in resisting salinity stress and promoting plant growth (Halo et al. 2015). LK11 also contains Na^+^/H^+^ antiporters (*nha*) that have also been shown to alleviate salinity stress (Epstein 2003).

### PGPR fitness against oxidative stress in *Sphingomonas* sp. LK11

Plants use various strategies to protect themselves from numerous viral, bacterial, and other threats. These strategies include the formation of reactive oxygen species (ROS; superoxide, hydroxyl radical, and hydrogen peroxide), phytoalexins, and nitric oxide (HammondKosack and Jones 1996; Zeidler et al. 2004). Aerobic organisms utilize various enzymes and antioxidants to manage oxidative stress resulting from the detrimental byproducts of aerobic respiration (Cabiscol et al. 2000; Lushchak 2001).

The LK11 genome encodes genes to protect itself during the activation of plant defense mechanisms; such genes encode glutathione S-transferase (locus AV944_12110, locus AV944_13280, and locus AV944_05350), glutathione peroxidases (locus AV944_12175), superoxide dismutases (SODs; locus AV944_13570 and locus AV944_06030) and glutathione-disulfide reductase (locus AV944_15970). Furthermore, the LK11 genome contains five genes encoding different catalases (locus AV944_17575) and eight genes encoding peroxidases. Genes encoding three peroxiredoxins and two glutaredoxins were also identified. As endophytic bacteria can mitigate oxidative stress, they could strengthen plant defenses against abiotic stress-induced ROS generation (Khan et al. 2017). This is also in agreement with a previous study where LK11 counteracted sodium chloride-induced ROS generation by increasing the activity of catalase, superoxide dismutase, and reduced glutathione (Halo et al. 2015).

### Cold shock and heat shock proteins in *Sphingomonas* sp. LK11

Under different environmental conditions, some bacteria can regulate cold shock and heat shock protein levels. The cold shock protein family comprises small, structurally related, and highly conserved nucleic acid-binding proteins that appear to contribute significantly to the management of numerous microbial physiological processes (Ermolenko and Makhatadze 2002). These proteins are extensively distributed among prokaryotes and are frequently encoded through differentially regulated, multiple gene families (Graumann and Marahiel 1998; Phadtare 2004). The LK11 genome contains the cold shock protein genes *cspA* and *cspB* (locus AV944_00095 and locus AV944_14525, respectively) and the heat shock protein genes *dnaJ* and *dnaK* (locus AV944_15200 and locus AV944_15205, respectively), *grpE* (locus AV944_15230), *hrcA* (locus AV944_15235), and *rpoH* (locus AV944_13700). These genes have been linked to the modulation of cold and heat adaptive functions. The presence of these genes further confirms our previous results where chaperone and L10 family of ribosomal proteins were significantly upregulated in response to cadmium-induced toxicity (Khan et al. 2014).

### Heavy metal resistance and improving phytoremediation strategies

**Fig. 3:**
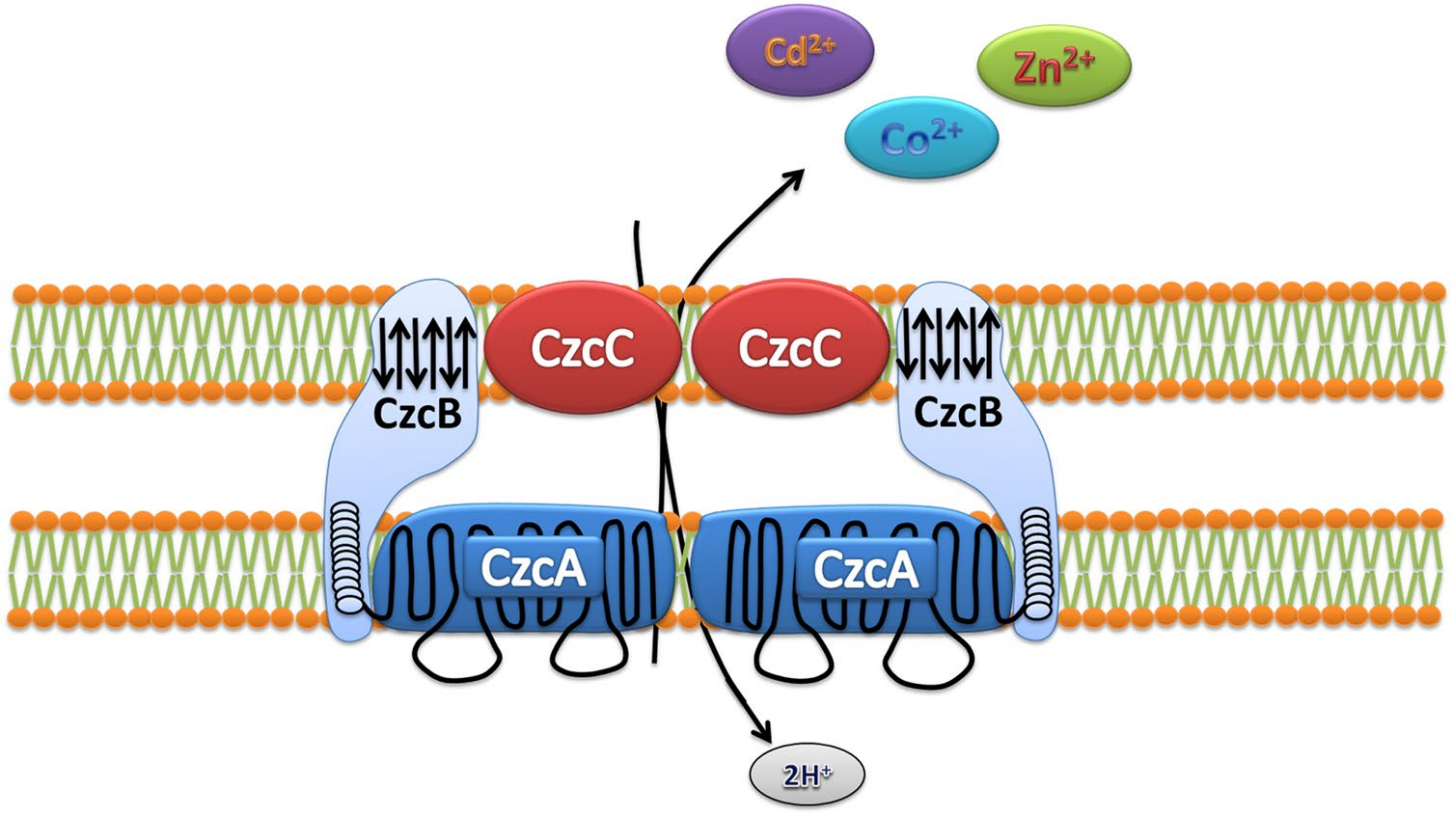
Proposed model for the *czc* efflux system in LK11 as suggested by Ludo Diels and adopted by Simon Silver (1996). It has been reported that CzcC is a cell wall “outer” membrane protein, CzcA is an “inner” plasma membrane transport protein, while CzcB is a membrane fusion protein that extends through both membranes

Many bacterial species possess mechanisms that make them resistant or tolerant to heavy metals (Diels et al. 1995; Ji and Silver 1995; Kunito et al. 1996). Our analysis of the LK11 genome revealed the presence of a *czc* operon in the chromosome and plasmids. The *czc* operon was found comprised of three structural genes, *czcA, czcB*, and *czcC*, as well as two regulatory genes, *czcD* and *czcR* (Table 3). This operon was previously found to confer resistance to three heavy metals, namely cobalt, zinc, and cadmium (Kunito et al. 1996; Nies 1995; Silver and Phung 1996). Various models of the *czc* efflux system have been proposed for different bacteria (Nies 1992). The most commonly occurring model was found in the LK11 genome where the efflux system exists as a dimmer (Fig. 3); this model was suggested by Diels et al. (1995) and was adopted by Silver (1996). Furthermore, the arsenic resistance genes *arsB* and *arsC* were found on the LK11 chromosome. Previous studies have shown that *arsB* and *arsC* encode arsenate reductase and aid in arsenite efflux transport (Duan et al. 2013).

**Table 3:** Genes potentially involved in metal resistance in the LK11 genome

Gene	Locus	Product
*czcA*	AV944_17755	Cobalt/zinc/cadmium resistance protein CzcA
*czsB*/*cusB*	AV944_17760	Cobalt/zinc/cadmium efflux RND transporter, membrane fusion protein, CzcB family
*czcC*	AV944_17765	Heavy metal RND efflux outer membrane protein, CzcC family
*czcD*	AV944_17715	Cobalt/zinc/cadmium resistance protein CzcD
*czcR*	AV944_17775	Cobalt/zinc/cadmium resistance protein CzcD
*hmrR*	AV944_17780	Transcriptional regulator, MerR family
*cueA*	AV944_17735	Copper-translocating P-type ATPase (EC 3.6.3.4)
*catalase hpII*	AV944_17575	Catalase related to oxidative stress
*copA*	AV944_17735	Multi-copper oxidase
*copB*	AV944_00295	Copper resistance protein
*copC*	AV944_14760	Copper homeostasis
*arsB*	AV944_04475	Arsenic efflux membrane protein
*arsC*	AV944_07460	Arsenate reductase

In addition, the LK11 genome also carries genes involved in copper resistance on its chromosome and plasmids (Table 3). Most of the bacterial genes that confer copper resistance are carried on plasmids and are organized in operons (Dupont et al. 2011; Magnani and Solioz 2007; Wei et al. 2009). It has been shown that copper resistance is encoded by the *copA, copB, copC*, and *copD* genes in various bacteria (Mellano and Cooksey 1988; Voloudakis et al. 2005). During genome analysis, we found that LK11 possess *copA*, a gene that encodes multi-copper oxidase; this gene is one of the main genetic elements involved in copper resistance in Gram-negative bacteria (Lejon et al. 2007; Nies 1999; Rensing and Grass 2003). Multi-copper oxidase is considered a marker gene for copper-resistant bacteria (Voloudakis et al. 2005). Moreover, plasmid 1 of LK11 contains the *cueA* gene (Table 3), which encodes a copper-transporting P-type ATPase for copper homeostasis. This gene is found in other bacteria especially in copper-resistance bacteria (Magnani and Solioz 2007). Previously, the plasmid pMOL28 of *Cupriavidus metallidurans* strain CH34 was found to confer resistance to nickel and cobalt toxicity (Liesegang et al. 1993; Tibazarwa et al. 2000) while plasmid pMOL30 conferred resistance against zinc, cadmium, cobalt (Nies and Silver 1989), and copper (Monchy et al. 2006). The presence of such genes in the *Sphingomonas* sp. LK11 plasmid coding for transport of metal ion supports its potential in microbe-assisted phytoremediation as previously reported (Khan et al. 2016b).

### Pan-genomic analysis of LK11

**Fig. 4:**
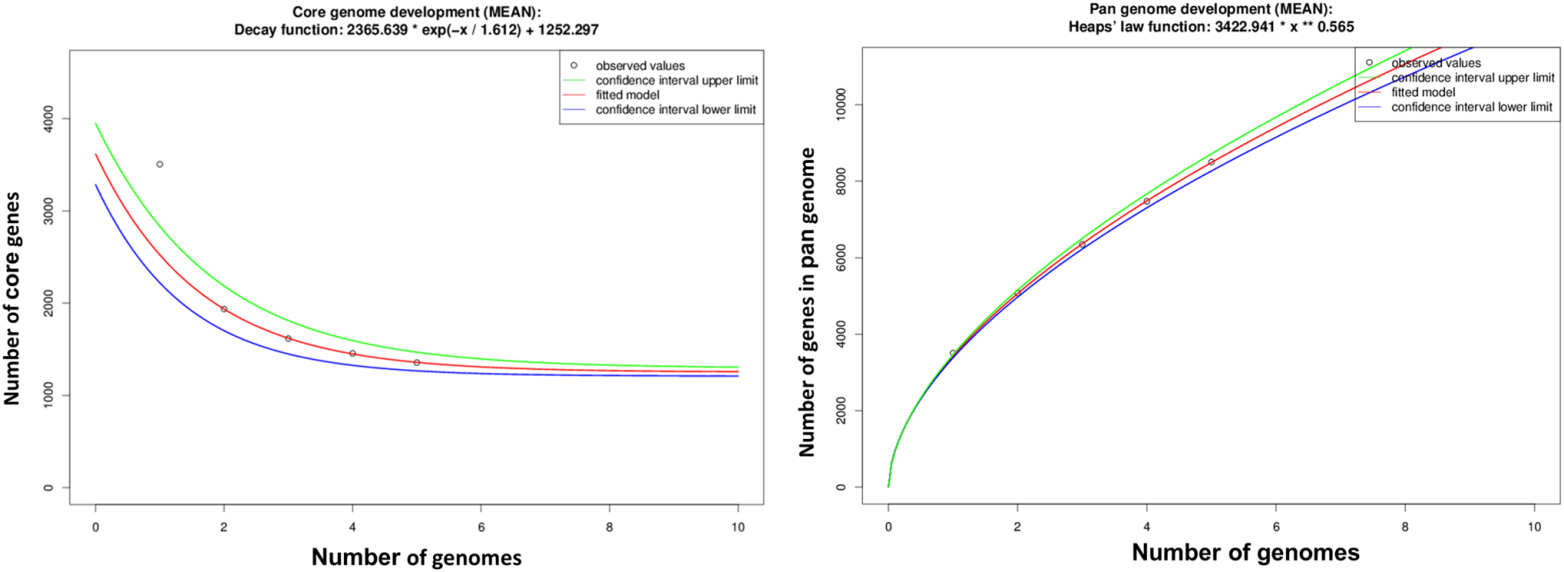
The number of gene clusters in the core and pan-genomes is plotted against the number of *Sphingomonas* spp. genomes sequenced

The pan-genome defines the complete complement of genes existing in a clade. In the present study, the full genomic sequences of LK11 and four other *Sphingomonas* species were used to investigate the core and pan-genome of sphingomonas genus. The core and pan-genome sizes were plotted against the number of genomes analyzed in this study. When additional genomes were added, the number of analogous gene clusters comprising the core genome dropped slightly, while the number of unique gene clusters in the pan-genome steadily increased. Extrapolation of the curve showed that the core genome contains a minimum of 1356 genes (95% confidence interval = 1209.4–1295.155) with the addition of *Sphingomonas taxi, Sphingomonas hengshuiensis, Sphingomonas* sp. MM1, and *Sphingomonas* sp. NIC1 genomes. The definitive number of shared genes in each genome deviates due to paralogs and duplicated genes (Fig. 4).

Furthermore, pan-genome analysis revealed that for every *Sphingomonas* species genome sequenced, an average of 1000 new genes were added to the pan-genome (Fig. 4). Likewise, the pan-genome curve showed that the representative species from genus *Sphingomonas* displayed an open pan-genome. The number of genomes examined were not enough to explain the complete gene sets and thus genomic sequencing of more *Sphingomonas* species is required to describe all genes of this genus. Furthermore, conserved genes are present across bacterial genomes within the same genus or species. A conserved fraction of these genes—specifically, those that are similar and found in all (or most) of the genomes within a given bacterial taxonomic group—is called the “core genome” of that group. The core genome can be identified on both the species and genus level (Leekitcharoenphon et al. 2012) and can be used to identify variable genes in a given genome (Adekambi et al. 2011). In general, conserved genes appear to evolve more slowly and can be used for establishing associations among various bacterial isolates (Urwin and Maiden 2003).

**Fig. 5:**
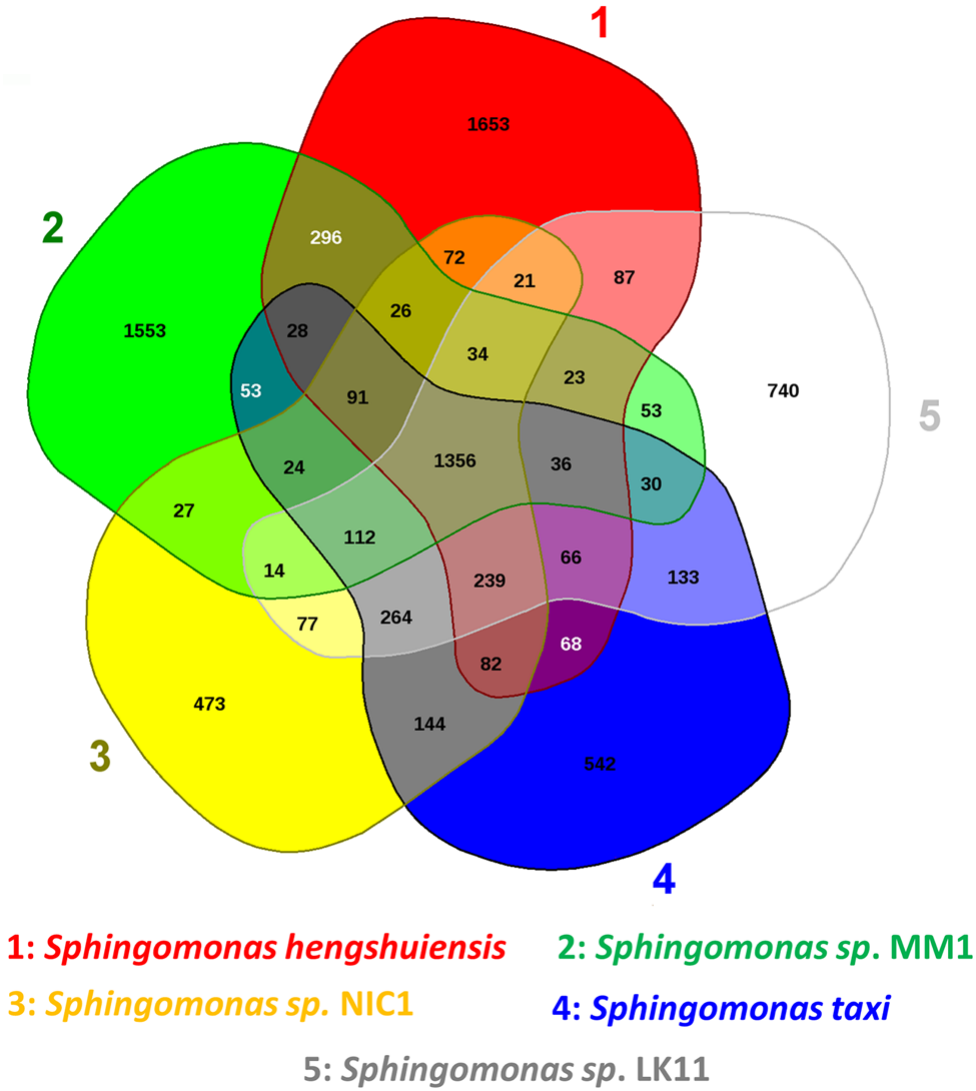
Venn diagram illustrating the orthologous gene complements of *Sphingomonas* sp. LK11 with *Sphingomonas* sp. MM1, *Sphingomonas* sp. NIC1, *Sphingomonas taxi*, and *Sphingomonas hengshuiensis*. Numbers in the outer circles represent the total number of unique genes identified in each genome while numbers in the center represent the number of orthologous sequences common to all five genomes

Additionally, the Venn diagram shows that 1356 genes are shared by all five *Sphingomonas* species analyzed. LK11 shares 53, 77, 133, and 87 genes exclusively with *Sphingomonas sp*. MM1, *Sphingomonas* sp. NIC1, *Sphingomonas taxi*, and *Sphingomonas hengshuiensis*, respectively (Fig. 5). The number of unique genes possessed by LK11, *Sphingomonas* sp. MM1, *Sphingomonas* sp. NIC1, *Sphingomonas taxi*, and *Sphingomonas hengshuiensis* were 740, 1553, 473, 542, and 1653, respectively (Fig. 5). Finally, the unique genes possessed by LK11 mostly encode hypothetical proteins and their GC content ranges from 48.6 to 75.1% with an average of 65% (Table S3). These unique genes include glutaredoxin-related (locus AV944_14990 and AV944_14990) and thioredoxin-related (locus AV944_10100) genes, which may be responsible for maintaining a cellular redox environment and may control oxidative stress responses in LK11 as previously reported (Zeller and Klug 2006). In addition, the genome of LK22 also includes the *arsR* gene family, which is a transcriptional regulatory protein class known to counter stress generated by heavy metal toxicity. Furthermore, TonB-dependent transporter-related genes were also found in LK11. TonB-dependent transporters are bacterial outer membrane proteins that bind and transport nickel chelates, vitamin B_12_, and carbohydrates (Noinaj et al. 2010).

## Conclusions

The current study elucidates the growth-promoting characteristics and complete genetic makeup of *Sphingomonas* sp. LK11. LK11 produced different types of GAs in pure culture and significantly improved soybean plant growth by altering endogenous hormone levels. Similarly, sequencing and analysis of the LK11 genome support its role as a plant growth-promoting bacterium, prompting further research. Complete genome sequencing confirmed the presence of genes that are involved in plant growth-promoting traits; these include phosphate solubilization and H_2_S synthesis, which can improve the growth of associated plants. Moreover, biosynthesis pathways of trehalose and glycine betaine were found in the LK11 genome. A total of 8507 genes were identified in the *Sphingomonas* spp. pan-genome and 1356 orthologous genes were found to comprise the core genome. Utilization of this remarkably versatile PGPB may be an important eco-friendly alternative in improving phytoremediation strategies and crop growth under extreme environmental conditions.

### Nucleotide sequence accession numbers

The assembled and annotated sequences of LK11 (one chromosome and two plasmids) were deposited in GenBank with accession numbers CP013916–CP013918. The information was also submitted to the Genomes Online Database (Gs0118031). The strain was deposited in the International Collection of Microorganisms from Plants (ICMP) under the accession number ICMP 21288.

## Electronic supplementary material

Below is the link to the electronic supplementary material.

Supplementary material 1 (DOCX 13 KB)

Supplementary material 2 (DOCX 15 KB)

Supplementary material 3 (XLS 99 KB)
