# Supplementary material for: Complete genome sequencing and analysis of endophytic Sphingomonas sp. LK11 and its potential in plant growth
Source: 3 Biotech. 2018 Aug 28;8(9):389. doi: 10.1007/s13205-018-1403-z (PMC6111035; doi:10.1007/s13205-018-1403-z)
Supplement: Supplementary file 1 — Supplementary material 1 (DOCX 13 KB) [file 13205_2018_1403_MOESM1_ESM.docx]

**Table S1. Genome assembly statistics**

| Number of bases | 1,003,181,780 |
| --- | --- |
| Number of reads | 84,384 |
| N50 read length | 15,917 |
| Mean read length | 11,888 |
| Average reference consensus concordance | 99.99% |
| Number of contigs | 3 |
| Average Reference Length | 1,312,439 |
| Average Reference Bases Called | 100% |
| Average coverage | 144.97 |
