# Supplementary material for: Complete genome sequencing and analysis of endophytic Sphingomonas sp. LK11 and its potential in plant growth
Source: 3 Biotech. 2018 Aug 28;8(9):389. doi: 10.1007/s13205-018-1403-z (PMC6111035; doi:10.1007/s13205-018-1403-z)
Supplement: Supplementary file 2 — Supplementary material 2 (DOCX 15 KB) [file 13205_2018_1403_MOESM2_ESM.docx]

**Table S2. Functional categories for the clusters of orthologous groups of proteins (COGs) of LK11**

|  | **Name** | **Count** | **Proportion** |
| --- | --- | --- | --- |
| E | Amino acid transport and metabolism | 185 | 6.98% |
| G | Carbohydrate transport and metabolism | 166 | 6.26% |
| D | Cell cycle control, cell division, chromosome partitioning | 31 | 1.17% |
| N | Cell motility | 75 | 2.83% |
| M | Cell wall/membrane/envelope biogenesis | 169 | 6.37% |
| H | Chromatin structure and dynamics | 1 | 0.04% |
| V | Coenzyme transport and metabolism | 147 | 5.54% |
| C | Defense mechanisms | 62 | 2.34% |
| W | Energy production and conversion | 168 | 6.33% |
| S | Extracellular structures | 17 | 0.64% |
| R | Function unknown | 178 | 6.71% |
| P | General function prediction only | 213 | 8.03% |
| U | Inorganic ion transport and metabolism | 162 | 6.11% |
| I | Intracellular trafficking, secretion, and vesicular transport | 50 | 1.89% |
| F | Lipid transport and metabolism | 118 | 4.45% |
| O | Nucleotide transport and metabolism | 50 | 1.89% |
| A | Post-translational modification, protein turnover, chaperones | 56 | 2.11% |
| L | RNA processing and modification | 134 | 5.05% |
| Q | Replication, recombination and repair | 114 | 4.3% |
| T | Secondary metabolite biosynthesis, transport, and catabolism | 59 | 2.22% |
| K | Signal transduction mechanisms | 165 | 6.22% |
| J | Transcription | 155 | 5.84% |
|  | Translation, ribosomal structure, and biogenesis | 177 | 6.67 |
|  | Not in COG database | 1,351 | 36.13% |
